# Retention on Buprenorphine for Opioid Use Disorder in Justice-Involved Individuals: A Retrospective Cohort Study

**DOI:** 10.3390/bs16010122

**Published:** 2026-01-15

**Authors:** Andrea Yatsco, Francine R. Vega, Audrey Sarah Cohen, Marylou Cardenas-Turanzas, James R. Langabeer, Tiffany Champagne-Langabeer

**Affiliations:** Center for Behavioral Emergency & Addiction Research, McWilliams School of Biomedical Informatics, The University of Texas Health Science Center, 7000 Fannin Street, Ste 600, Houston, TX 77030, USA; andrea.j.yatsco@uth.tmc.edu (A.Y.); maria.cardenasturanzas@uth.tmc.edu (M.C.-T.); james.r.langabeer@uth.tmc.edu (J.R.L.); tiffany.champagne@uth.tmc.edu (T.C.-L.)

**Keywords:** opioid use disorder, buprenorphine, criminal justice involvement, behavioral health, MOUD retention

## Abstract

Criminal justice system (CJS) involvement is common among individuals with opioid use disorder (OUD), yet limited research examines retention in medications for OUD (MOUD) within community settings. This study assessed whether CJS involvement predicted retention on buprenorphine/naloxone and explored related demographic and clinical factors. A retrospective cohort included adults (n = 367) enrolled in a low-barrier outpatient MOUD program in Texas (January 2022–April 2024). CJS involvement was identified from program records. Retention was measured as the number of continuous days with buprenorphine/naloxone prescriptions. Analyses used univariate tests, logistic regression, and nonparametric kernel regression. Nearly one-quarter (24.8%) were CJS-involved. Retention at 180 days was similar between CJS and non-CJS groups (38%). CJS participants initiated substance use earlier and reported higher heroin and injection drug use. Behavioral health sessions were associated with both CJS involvement (OR = 1.10, *p* ≤ 0.001) and longer retention (β = 10.81 days/session, *p* = 0.001). With comprehensive, low-barrier services, individuals involved with CJS achieved MOUD retention comparable to their peers. Early behavioral health engagement was a strong predictor of retention, suggesting a key intervention point to enhance outcomes and advance equity for justice-involved populations.

## 1. Introduction

The convergence of the opioid crisis and criminal justice involvement has contributed to a growing public health emergency with unprecedented levels of global fatal overdoses. The National Academies have emphasized the justice system as an essential component in the fight against the opioid epidemic (National Academies of Sciences, Engineering, and Medicine, 2019). While the effectiveness of evidence-based treatments such as medications for opioid use disorder (MOUD) is well established (Hazelton et al., 2025; Kennedy et al., 2022; Krawczyk et al., 2021), their utilization in the United States remains suboptimal, particularly among justice-involved populations (Stahler et al., 2022). Factors that influence positive outcomes and/or adverse events while receiving substance treatment, including criminal justice status, are still not well understood. Stigma and paternalism within the criminal justice system may influence an individual’s choice to seek treatment (Debbaut, 2022).

Despite policy momentum, fewer than 6% of justice-involved individuals receive community-based MOUD (Guastaferro et al., 2022). While incarceration may briefly curb substance use, substance-related concerns often endure beyond release (Tangney et al., 2016). This research highlights a critical gap in understanding how justice-involved individuals navigate re-entry into outpatient MOUD treatment, and what outcomes they experience.

### 1.1. Medication for Opioid Use Disorder in the Criminal Justice System

MOUD is the gold-standard treatment for OUD and has been shown to significantly reduce the risk of future overdose by 59% over a 12-month period (adjusted hazards ratio, HR = 0.41, 95% CI, 0.31–0.55) compared to no intervention (Wakeman et al., 2020). There are three medications approved for treatment of OUD by the U.S. Food and Drug Administration: methadone, a full opioid receptor agonist; naltrexone, an opioid antagonist; and buprenorphine, a partial opioid agonist that is commonly combined with an antagonist naloxone to both decrease diversion and abuse (Raisch et al., 2002). Methadone requires daily dosing at an opioid treatment program, whereas buprenorphine or buprenorphine/naloxone can be administered or dispensed as take-home formulations through retail pharmacies, offering greater prescribing flexibility and access without daily clinic visits (Substance Abuse and Mental Health Services Administration [SAMHSA], 2021, 2024). Within jail and prison populations, systematic reviews have shown that buprenorphine has similar costs and overdose risk profiles to methadone (Wakeman et al., 2020), with significantly lower regulatory and logistical costs (Moore et al., 2019). In one state prison system in Massachusetts, buprenorphine showed the highest level of usage and was associated with better long-term outcomes post-release (Bovell-Ammon et al., 2024).

MOUD remains underused among justice-involved individuals, who face high rates of OUD, overdose, financial instability, and other adverse outcomes, including elevated post-incarceration overdose risk (Krawczyk et al., 2017; Langabeer et al., 2024; Binswanger et al., 2016; Grella et al., 2021). Long-term MOUD improves abstinence and reduces overdoses, emergency department visits, arrests, and serious opioid-related acute-care events. It is also recommended by multiple clinical guidelines (Kraus et al., 2011; Wakeman et al., 2020; Dever et al., 2024). However, referrals from the justice system remain low, and those who obtain treatment are understudied. Access is further limited by structural, attitudinal, and logistical barriers, discontinuity of care during incarceration, and stigma or limited MOUD knowledge among justice personnel (Staton et al., 2023; Booty et al., 2023; Finlay et al., 2016). Despite its proven effectiveness, initiating and retaining individuals in MOUD remains a major challenge.

### 1.2. Treatment Utilization

Existing research on justice-involved individuals and MOUD largely focuses on incarcerated populations or relies on administrative data that overlook clinical engagement. Criminal justice costs related to opioid overdose, misuse, and dependence exceed USD 14 billion annually, driven primarily by enforcement rather than treatment (Florence et al., 2021). Although roughly 6 million justice-involved individuals were referred to substance use treatment in the past year (Rowell-Cunsolo & Bellerose, 2021), studies often fail to distinguish treatment access from actual utilization, and few examine justice-involved patients enrolled in outpatient MOUD programs. Much of the literature depends on commercial or public insurance claims, limiting generalizability and insight into patient engagement (Zhang et al., 2022). Consequently, few studies have assessed comprehensive MOUD treatment among justice-involved populations. This study addresses that gap by evaluating whether justice involvement is associated with differences in MOUD retention in a community-based outpatient setting, adjusting for demographic and clinical characteristics, with implications for improving care quality and engagement.

## 2. Materials and Methods

### 2.1. Study Design, Study Sample

A retrospective cohort study was conducted to assess retention on buprenorphine/naloxone among individuals enrolled in a community-based outpatient program between January 2022 and April 2024. Participants were adults enrolled in an outpatient program in Houston, Texas, providing rapid access to buprenorphine/naloxone and comprehensive behavioral support. This specific medication combination was the sole medication available in this program, which was chosen largely because of lower cost, logistics, and regulatory requirements compared to methadone and naltrexone. For individuals currently on MOUD elsewhere, they remained on MOUD and did not see a medical provider, unless opted to transition to buprenorphine, and they were excluded from this analysis.

Referral sources for study participants included the pre-hospital network of providers who stabilize and transport patients to medical facilities, such as Emergency Medical Services (EMS), overdose follow-ups, emergency departments, the criminal justice system, and self-referrals (Langabeer et al., 2021). MOUD (buprenorphine/naloxone) was available to all participants regardless of justice status, and access to behavioral services was not contingent on MOUD uptake. Patients first meet with a peer support specialist for a motivational interview to assess their readiness for change. At enrollment, all individuals were offered the opportunity to see a prescribing clinician, such as a medical doctor, nurse practitioner, or physician assistant, for an initial prescription of buprenorphine/naloxone. Dosage was based on clinical presentation of cravings and best practice prescribing guidelines, with the standard starting dosage of 8 mg buprenorphine/2 mg naloxone. Patients were encouraged to begin medications, but they were allowed to participate in their recovery journey by selecting from a variety of other services, including individual counseling from a licensed clinician, access to recovery support peer specialists from peer recovery coaches, group counseling and peer support groups, and general case management including risk/need assessments and navigation to additional resources not available internally. Inclusion criteria for this study required participants to have filled at least one prescription for buprenorphine/naloxone post-enrollment to analyze retention for those selecting a MOUD pathway. Ethical approval for this intervention was obtained from the university’s Institutional Review Board, and all participants provided written informed consent.

Criminal justice system (CJS) involvement was determined through a review of five program documentation fields on the biopsychological intake form: incarceration outcomes, new court case documentation, closed court case documentation, staff support in court, and documentation of current active legal case with the justice system. Participants were classified as CJS-involved if any of these indicators were positive at any point. A participant was classified as non-CJS involved if all five fields were negative.

### 2.2. Study Variables

The primary outcome was MOUD retention, defined as the number of days between the first and last continuous prescriptions for buprenorphine/naloxone, verified via the Texas Prescription Drug Monitoring Program (PMP). In accordance with best practice, an initial prescription of 7 days was provided to patients to ensure proper usage and tolerance to buprenorphine (Substance Abuse and Mental Health Services Administration [SAMHSA], 2021; U.S. Food and Drug Administration [FDA], 2021; National Alliance of Advocates for Buprenorphine Treatment [NAABT], n.d.; Hughes et al., 2024). Subsequent prescriptions were of a 30-day duration. A standard maximum prescription for buprenorphine/naloxone in the United States is 30 days. Ongoing prescriptions were considered continuous if refilled within 45 days of the previous prescription. The 45 days allowed for discrepancies in prescription pick-up timelines that may not have represented a true discontinuation of medication. This study did not capture engagement with other FDA-approved medications for OUD, including methadone or naltrexone. The secondary outcome involved a comparison of demographic and clinical characteristics between individuals with and without CJS involvement.

### 2.3. Moderating and Control Variables

Demographic variables included age, gender, race, ethnicity, veteran status, insurance status, and housing status. Clinical variables included age of opioid initiation, intravenous drug use history, primary opioid type (prescription or illicit), co-occurring substance use, overdose history, and prior MOUD experience. Behavioral engagement was assessed by the number of sessions with counselors, peers, or groups within the first 30- and 90 days post-enrollment. The total number of sessions served was a proxy for program participation.

### 2.4. Statistical Analysis

Frequencies, means, medians, and proportions were calculated to describe the sample. Univariate comparisons were conducted using t-tests for normally distributed variables, Kruskal–Wallis equality of population rank tests for non-normally distributed variables, and Fisher’s exact test for categorical variables. Pearson correlations were used for associations between continuous variables. For the univariate analyses of the two study outcomes, we tested multiple hypotheses, and the Bonferroni correction was applied to control for Type I error, indicating significance (*p* ≤ 0.001).

Because MOUD days were non-normally distributed and heteroskedasticity of the observations was present, a nonparametric kernel regression model (Epanechnikov kernel function) was used to evaluate predictors of retention (Cattaneo & Jansson, 2018; Li et al., 2003; Li & Racine, 2004). This model included covariates with an univariate significance level of *p* ≤ 0.05, with bootstrapping to estimate confidence intervals. Variables in the model included housing status, intravenous drug use, opioid type, MOUD history, and program engagement. Considering that the variables “primary opioid taken was prescription” and “primary opioid taken was illegal” were significantly and inversely correlated, we included in the model only the former, as it explained more variance and resulted in a better goodness-of-fit. CJS involvement was included as a priori variable of interest.

An exploratory binary logistic regression model was constructed to identify predictors of our secondary outcome, CJS involvement, including covariates with *p* ≤ 0.10 in univariate testing. The model goodness-of-fit was assessed using the Hosmer and Lemeshow test at χ^2^ = 11.85 (df = 8), *p* = 0.16. The CJS model included 341 observations and had an R-squared of 0.37. The use of the Bonferroni formula in this model indicated significance at a *p* ≤ 0.006. All analyses and graphical outputs were conducted using Stata/IC version 15.0 (Stata, 2017).

## 3. Results

### 3.1. Sample Characteristics

A total of 540 charts of adult participants in the program were reviewed. Participants had enrolled between 3 January 2022, and 30 April 2024. Ten (1.8%) participants were excluded from the analysis because they entered the program before the earliest date retrievable from the PMP database. Among the 530 participants, 163 (30.8%) were excluded because they did not have at least one prescription filled per PMP. A total of 367 were included in this analysis, and we observed the mean (sd) age of this group was 35.2 (9.7) years.

### 3.2. MOUD Retention

Of the 367 participants, at the cutoff point of 180 days in the program, 133 (36.2%) remained on MOUD, 35 of which (10%) were involved in the CJS; equating to 38% retention at 180 days of the initial justice-involved group (n = 91) as seen in Table 1. We found that involvement in the CJS was significantly associated with the number of sessions served during the first 90 days. Participants involved in the CJS had a median of 12, with interquartile range (IQR) of (4–20) sessions served, while those not involved in the system had only a median (IQR) of 3 (0.5–8) sessions (*p* ≤ 0.0001).

There were some notable differences in proportions of key substances used and clinical history between individuals with CJS involvement (n = 91) and those without (n = 276). Individuals involved in the CJS reported an earlier mean age of substance use initiation (18.2 years vs. 21.1 years; *p* = 0.007) and a higher proportion of use of heroin as the primary illicit drug (67.0% vs. 48.5%; *p* = 0.002).

### 3.3. Modeling MOUD Retention Outcomes

Retention in the program was measured by days using MOUD. For all the participants, we found this variable had a non-normal distribution with an unweighted mean (sd) of 203.9 (261) days and an unadjusted median (IQR) of days on MOUD at 61 (7–349) days. Table 2 highlights the univariate analysis which indicated a significant and positive correlation between the total number of sessions served in the first 90 days in the program and days on MOUD (*p* ≤ 0.001).

### 3.4. Characteristics by Justice Involved Status

The exploratory multivariable binary logistic regression model included as dependent variable involvement in the CJS (yes/no) and as independent covariates: gender, race, age at first drug use, use of IV drugs, primary drug taken was prescription, primary drug taken was illegal, type of illicit drug consumed was heroin, first time on MOUD, and total number of sessions served in first 90 days in program. The model had satisfactory goodness of fit, and the number of sessions served was the only independent predictor found. Each additional session within the first 90 days was associated with a 10% increase in the odds of criminal justice involvement (OR = 1.10, 95% CI = (1.07 to 1.14), *p* ≤ 0.001), suggesting individuals involved in CJS may have engaged more intensely early in treatment (Table 3).

#### Multivariate Analysis CJ Involvement and Days on MOUD

Next, we constructed an exploratory multivariable nonparametric kernel regression model with the total days in MOUD as the dependent variable. The covariates included were: CJS involved, housing, history of use of IV drugs, primary opioid taken was prescription, first time on MOUD, total number of sessions served in the first 90 days in the program, primary opioid prescribed was hydrocodone, and primary other substance consumed was methamphetamine. The weighted mean days on MOUD in this group of patients was 201 days (95% CI = (172 to 234), *p* = 0.001). Three covariates had a significant impact on the days on MOUD. Compared to participants who had used MOUD before, first-time users of MOUD had significantly fewer days on MOUD (−79.72, 95% CI = (−138.40 to −27.21), *p* = 0.001). Compared to those whose primary other substance consumed was not methamphetamine, participants who used this substance had significantly fewer days on MOUD (−104.21, 95% CI = (−165.03 to −45.73), *p* = 0.001). The number of sessions served in the first 90 days in the program was an independent predictor of days on MOUD; an increase in one activity during this period in the program would increase 10.81 days the total days on MOUD (10.81, 95% CI = (5.55 to 15.67), *p* = 0.001) shown in Table 4.

### 3.5. Predictors of Days on MOUD

**Figure 1:**
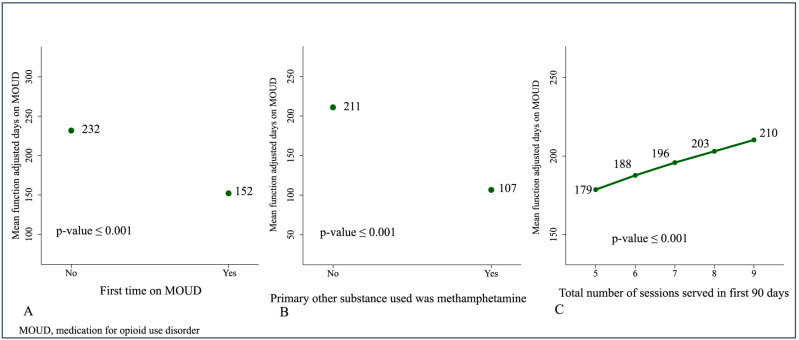
Predictive margins of adjusted mean function of days in MOUD by (**A**) First time on MOUD, (**B**) Primary other substance used was methamphetamine, and (**C**) Total number of sessions served in the first 90 days of the program.

The predictive margins of the three independent predictors observed are depicted in Figure 1. Assuming the rest of the covariates stay at mean values, the analyses of margins indicated the adjusted mean days predicted for those who used MOUD for the first time (A) was 152 (95% CI = (111 to 187), *p* ≤ 0.001) vs. 232 days (95% CI = (194 to 269), *p* ≤ 0.001). Participants who had not used methamphetamines had a predicted adjusted mean of 211 days (95% CI = (184 to 241), *p* ≤ 0.001), while participants who did use methamphetamines had an adjusted mean of only 107 days (95% CI = (57 to 170), *p* ≤ 0.001) (B). Increasing the number of sessions served during the first 90 days in program from five (the median number of the study group) to seven sessions resulted in an increment of 17 days (C), from a predicted adjusted mean of 179 days (95% CI = (150 to 215), *p* ≤ 0.001) at five sessions to an adjusted mean of 196 days (95% CI = (164 to 236), *p* ≤ 0.001) when seven sessions were served. See Figure 1A–C.

## 4. Discussion

Retention in MOUD treatment remains a national challenge (Timko et al., 2016). Our results support prior research which demonstrates that many individuals discontinue treatment before the six-month mark (Kennedy et al., 2022). However, the weighted mean within the cohort in this study reached 201 days. This is an encouraging outcome in comparison to national trends. The total number of sessions served in the first 90 days in the program served as a proxy of engagement of the participants with extended services (e.g., peer recovery coaching, individual therapy, group support). This outcome was significantly associated with increased retention in the treatment program which supports prior evidence that MOUD, when paired with comprehensive, individualized services, produces more durable outcomes (Ahmed et al., 2022; Pivovarova et al., 2023; Zhang et al., 2022). We also observed that first-time adopters of MOUD and the concurrent consumption of methamphetamine with opioids significantly reduced the retention in the program.

We did not find CJS to be an independent determinant of days on MOUD. However, the criminal justice relationship may be more nuanced. This study showed some differences in substance use and MOUD history between groups. Individuals involved in the CJS experienced more severe substance use profiles, marked by higher proportions of earlier initiation of opioids, heroin use and injection/intravenous as the reported route of administration. A higher proportion of individuals in the CJ system were first-time MOUD participants, and the predictive model for retention highlighted that, compared to previous use of MOUD, first-time MOUD utilization was significantly associated with a lower weighted mean function of days in MOUD.

Notably, engagement in behavioral services was associated with increased odds of being CJS involved. This may suggest that justice-involved individuals, who are often under legal or social pressure to demonstrate recovery progress, are more motivated or mandated to engage with these services, independent of their retention timeline on MOUD. While mandated treatment can be a factor for retention, our justice variable comprised justice involved individuals captured in different phases of the justice process with mandated treatment not assumed or probable for the entire sample. Additionally, research has shown that mandated treatment does not necessarily result in perceived coercion (Hachtel et al., 2019) and can lead to positive outcomes, however engagement, retention, and completion rates in mandated samples remains low (Coviello et al., 2013). The high engagement rates for the justice subgroup in this study is noteworthy; however, since mandated treatment was not explicitly controlled for in this sample results should be cautiously interpreted.

A final finding worth highlighting is the potential impact of polysubstance use, particularly concurrent stimulant use, on treatment retention. In this analysis, individuals reporting methamphetamine use experienced significantly lower retention. A pattern of concurrent opioid and stimulant use mirrors national data from the “fourth wave” of the opioid crisis (Ciccarone, 2021), which makes the outcomes lower retention particularly concerning given the current landscape. With no FDA-approved pharmacotherapy for stimulant use disorder, this subgroup remains vulnerable and underserved. Future research into this area could explore if stimulant use serves to moderate or mediate the relationship with treatment outcomes.

The convergence of opioid use disorder and justice system involvement persists across the continuum, from incarceration to specialty courts and community supervision. Justice-involved individuals remain significantly less likely to access MOUD, with referral rates consistently below 6% (Guastaferro et al., 2022; Krawczyk et al., 2017). In our study, individuals with prior exposure to MOUD exhibited significantly better retention than first-time participants, many of whom were justice-involved. The outcome of MOUD attempts is in line with current research that suggests that abstinence from opioids often requires multiple attempts before successful (Fontes et al., 2025). This sample uncovered a majority of first time MOUD adopters as justice involved, which may signal increasing access to evidence-based treatment for justice populations. It also underscores the critical role of education and targeted support for first-time MOUD recipients, both for justice involved individuals and the staff working in justice settings. A lack of existing research comparing first time MOUD experiences relevant to justice status also marks this perspective as particularly rich for further analysis. Although all participants in this study had access to MOUD, uptake among justice-involved individuals was still incomplete, indicating ongoing gaps in utilization.

### Limitations

This study focused solely on buprenorphine, one of three FDA-approved MOUDs, as it was the only medication intervention provided in this treatment program. Some individuals classified as non-MOUD may have received methadone or naltrexone elsewhere. Additionally, results in this study should be viewed in the context of other buprenorphine outcomes research and not generalized to all MOUD medications/treatment. Comparisons between MOUD and non-MOUD pathways were beyond the scope of this study. As a retrospective, non-randomized study drawn from a voluntary treatment program, findings may be influenced by self-selection and are not generalizable beyond this specific setting. MOUD was offered to all participants, but engagement varied, with some opting into non-pharmacological services only. Program structure and community context may limit applicability to other regions.

## 5. Conclusions

Decades of advocacy have established MOUD as a first-line, evidence-based treatment. Yet several studies (Samples et al., 2018; Agbese et al., 2020; Hasan et al., 2021) have shown early discontinuations despite Centers for Medicare and Medicaid quality metric recommendation of a treatment episode duration of a minimum of six months (Centers for Medicare and Medicaid, 2024). Although excluding individuals who have not initiated buprenorphine may create selection bias and limit generalizability, focusing on individuals who have filled at least one prescription addresses the question of retention as opposed to initiation, which is behaviorally distinct with different health determinants. Efforts to shift cultural perceptions and eliminate stigma must continue to ensure that justice-involved individuals are not excluded from these lifesaving therapies. Given the scant research on justice status as an available variable in treatment comparisons, this study expands the knowledge surrounding access to comprehensive, coordinated treatment services for justice populations. Our findings provide evidence from a within program sample that justice status was not a significant independent predictor of retention outcomes, increasing confidence that justice-involved individuals can achieve similar retention outcomes to their non-justice-involved peers. The strong partnerships between justice stakeholders and the outpatient program in this study likely contributed a statistically meaningful sample size of justice-involved clients to analyze. Such collaboration is also key to reducing systemic barriers to care for justice populations (Yatsco et al., 2020a, 2020b). Investing in such programs and incorporating greater understanding about the overlap of justice and treatment is a public health and healthcare access imperative.

While peer support services were available, their specific role in influencing justice-involved participants’ treatment outcomes warrant further study. The broader literature offers limited insight into the experiences of justice-involved individuals who do enter MOUD programs in community settings. Future research should explore why some justice-involved clients choose medication treatment while others do not, ideally through qualitative inquiry. Expanding justice-status data collection and replicating this analysis across diverse settings will be essential to deepen understanding and inform equitable MOUD implementation strategies. This study demonstrates that justice involvement does not independently predict buprenorphine retention, suggesting that justice-involved individuals can achieve comparable treatment outcomes when provided equitable access, underscoring the need for broader, more generalizable research to guide effective and stigma-free MOUD implementation.

## Figures and Tables

**Table 1:** Select demographic and clinical characteristics by involvement in the criminal justice system of participants prescribed MOUD (n = 367).

Demographics/Clinical Characteristic	Involved in the CJS N (%)	Not Involved in the CJS N (%)	Total N (%)	*p*-Value
Total	91 (24.8)	276 (75.2)	367 (100)	NA
Age, mean (sd)	33.8 (7.9)	35.6 (10.2)	35.2 (9.7)	0.13 *
Gender (Sex assigned at birth)				0.07 **
Men	57 (62.6)	141 (51.6)	198 (54.4)	
Woman	34 (37.4)	132 (48.4)	166 (45.6)	
Race				0.03 **
White	79 (87.8)	198 (77.3)	267 (80.1)	
Non-White	11 (12.2)	58 (22.7)	69 (19.9)	
Housing				0.89 **
Own/rent apartment or home	30 (33.0)	95 (34.4)	125 (34.1)	
Live with family or friends	47 (51.6)	144 (52.2)	191 (52.0)	
Homeless or other	14 (15.4)	37 (13.4)	51 (13.9)	
Insurance				0.62 **
No	59 (64.8)	169 (61.2)	228 (62.1)	
Yes	32 (55.2)	107 (38.8)	139 (37.9)	
Age at first use of substances, mean (sd)	18.2 (6.4)	21.1 (8.6)	20.4 (8.2)	0.007 *
History of use of IV drugs				0.02 **
No	34 (41.5)	148 (57.1)	182 (53.4)	
Yes	48 (58.5)	111 (42.9)	159 (46.6)	
Primary opioid taken was a prescription/pressed pills				0.09 **
No	55 (60.4)	138 (50.0)	193 (52.6)	
Yes	36 (39.6)	138 (50.0)	174 (47.4)	
Primary opioid taken was illicit				0.01 **
No	20 (22.0)	100 (36.2)	120 (32.7)	
Yes	71 (78.0)	176 (63.8)	247 (67.3)	
Type of primary opioid prescribed ^				
Oxycodone	27 (29.7)	99 (35.9)	126 (34.3)	0.31 **
Hydrocodone	23 (25.3)	80 (29.0)	103 (28.1)	0.59 **
Codeine	2 (2.2)	9 (3.3)	11 (3.0)	0.99 **
Morphine	1 (1.1)	6 (2.2)	7 (1.9)	0.99 **
Buprenorphine	2 (2.2)	2 (0.7)	4 (1.1)	0.26 **
Tramadol	1 (1.1)	6 (2.2)	7 (1.9)	0.99 **
Methadone	0	4 (1.4)	4 (1.1)	0.58 **
Hydromorphone	0	1 (0.4)	1 (0.3)	0.99 **
Type of primary illicit substances consumed ^				
Heroin	61 (67.0)	134 (48.5)	195 (53.1)	0.002 **
Fentanyl	34 (37.4)	77 (27.9)	111 (30.2)	0.11 **
Other non-prescribed opioids	2 (2.2)	11 (4.0)	13 (3.5)	0.53 **
Prior use of MOUD				0.02 **
Yes	29 (31.9)	127 (46.0)	156 (42.5)	
No	62 (68.1)	149 (54.0)	211 (57.5)	
Days on MOUD, median (IQR)	97 (20–379)	48 (7–320)	61 (7–349)	0.16 ^
Total number of sessions served at the 90th day in program, median (IQR)	12 (4–20)	3 (0.5–8)	5 (1–11)	≤0.0001 ***

CJS, Criminal Justice System; NA, not available; IV, intravenous drugs; MOUD, medication for opioid use disorder. * Student’s *t* test; ** Fisher’s Exact test; *** Kruskal–Wallis equality of population rank test; ^ Rows are independent of each other, so percentages in column do not add up to 100. Note: after applying the Bonferroni correction formula to decrease Type I error, a *p* ≤ 0.001 is significant.

**Table 2:** Days in MOUD by selected sociodemographic, clinical characteristics, and the total number of sessions served at 90 days, of participants in the study.

Characteristic	Total N (%)	Days in MOUD Median (IQR)	*p*-Value
Total	367 (100)	61 (7–349)	NA
Involved in the CJS			0.16 *
Yes	91 (24.8)	97 (20–379)	
No	276 (75.2)	48 (7–320)	
Age at entry to the program, mean (sd)	35.2 (9.7)	61 (7–349)	0.16 **
Housing			0.05 *
Own/rent apartment or home	125 (34.1)	107 (19–424)	
Live with family or friends	191 (52.0)	39 (7–258)	
Homeless or other	51 (13.9)	119 (7–338)	
Age at first drug use, mean (sd)	20.4 (8.2)	61 (7–349)	0.81 **
History of use of IV drugs			0.02 *
No	182 (53.4)	82 (11–419)	
Yes	159 (46.6)	35 (7–236)	
Primary opioid taken was a prescription			0.04 *
No	193 (52.6)	39 (7–282)	
Yes	174 (47.4)	102 (14–418)	
Primary opioid taken was illegal			0.03 *
No	120 (32.7)	120 (11–429)	
Yes	247 (67.3)	43 (7–274)	
Primary drug taken was other substance			0.09 *
No	275 (25.1)	77 (7–386)	
Yes	92 (74.9)	32 (7–234)	
First time on MOUD			0.02 *
No	211 (57.5)	106 (14–419)	
Yes	156 (42.5)	42 (7–226)	
Overdosed			0.17 *
Yes	226 (38.4)	49 (7–290)	
No	141 (61.6)	84 (8–419)	
Type of visit and MOUD prescription			0.08 *
Visited and prescribed MOUD by MP	343 (94.0)	51 (7–355)	
Not visited the MP but prescribed	22 (6.0)	158 (76–338)	
Total sessions served in the first 90 days in the program, median (IQR)	5 (1–12)	97 (19–386)	0.0001 **

* Kruskal–Wallis equality of population rank test, ** Pearson’s correlation. MOUD, medication for opioid use disorder; IQR, interquartile range; NA, not available; sd, standard deviation; IV, intravenous; MP, medical provider. Note: After applying the Bonferroni correction formula to decrease Type I error, a *p*-value ≤ 0.001 is significant.

**Table 3:** Multivariable exploratory binary logistic regression analysis of determinants of involvement in the Criminal Justice System of participants who were prescribed and used MOUD for one day or more.

Characteristic	OR	Std. Error	95% CI	*p*-Value
Gender				
Women	Reference			
Men	1.52	0.45	(0.85 to 2.72)	0.16
Race				
White	Reference			
Non-White	0.83	0.33	(0.37 to 1.83)	0.64
Age at first drug use	0.96	0.02	(0.91 to 0.99)	0.04
Use of IV drugs				
No	Reference			
Yes	0.98	0.35	(0.48 to 1.99)	0.95
Primary opioid taken was a prescription				
No	Reference			
Yes	0.63	0.23	(0.31 to 1.30)	0.21
Type of primary illicit substance consumed was heroin				
No	Reference			
Yes	1.36	0.49	(0.67 to 2.74)	0.39
First time on MOUD				
Yes	0.68	0.21	(0.36 to 1.26)	0.22
No	Reference			
Total sessions served in the first 90 days in the program				
	1.1	0.02	(1.07 to 1.14)	≤0.001

IV, intravenous drugs; MOUD, medication for opioid use disorder. Note: the model correctly classified 76.5% of the observations. The goodness-of-fit of the model measured with the Hosmer and Lemeshow test was χ^2^ = 11.85 (df = 8), *p* = 0.16. After applying the Bonferroni correction formula to decrease Type I error, a *p* ≤ 0.006 is significant.

**Table 4:** Multivariable exploratory nonparametric kernel regression analysis of determinants of days in MOUD in 367 participants in a program to treat opioid use disorder.

	Observed Estimate	95% CI	Bootstrap Std. Error	*p*-Value
Weighted mean of the days on MOUD	201.38	(172.22 to 233.52)	15.25	≤0.001
Covariates effects				
Involved in the CJS vs. not involved	−53.83	(−121.16 to 16.08)	36.27	0.14
Housing				
Living with family or friends vs. Own/rent apartment or house	−51.48	(−97.48 to −2.50)	24.52	0.04
Homeless or other vs. Own/rent apartment or house	−71.58	(−156.60 to 10.65)	43.08	0.1
History of use of IV drugs	−79.63	(−135.21 to −17.74)	30.28	0.009
Primary opioid taken was prescription	−45.51	(−125.27 to 30.99)	38.82	0.24
First time on MOUD	−79.72	(−138.40 to −27.21)	28.74	0.006
Total sessions served in the first 90 days in the program	10.81	(5.55 to 15.67)	2.64	0.001
Primary opioid prescribed was hydrocodone vs. not	63.44	(−13.22 to 144.88)	40.43	0.12
Primary other substance consumed was methamphetamine vs. not	−104.21	(−165.03 to −45.73)	30.43	0.001

CI, confidence interval; std., standard; MOUD, medication for opioid use disorder; CJS, criminal justice system; IV, intravenous; Note: Effect estimates are averages of derivatives for continuous covariates and averages of contrasts for factor covariates. After applying the Bonferroni correction formula to decrease Type I error, a *p* ≤ 0.006 is significant.

## Data Availability

The datasets presented in this article are not readily available because the data are part of an ongoing study.
